# Efficient Ultrasound-Assisted Synthesis of Chemically Supported Anionic Functional Group Ionic Liquids and Its Enhanced Adsorption Performance Towards Vanadium (V)

**DOI:** 10.3390/ma18061330

**Published:** 2025-03-18

**Authors:** Bo Chen, Shenxu Bao, Yimin Zhang, Jiahao Zhou, Wei Ding, Liuyi Ren, Siyuan Yang, Ye Zhang

**Affiliations:** 1 Key Laboratory of Green Utilization of Critical Non-Metallic Mineral Resources, Ministry of Education, Wuhan University of Technology, Wuhan 430070, China; rly1015@163.com (L.R.); siyuan.yang@whut.edu.cn (S.Y.); 2 School of Resources and Environmental Engineering, Wuhan University of Technology, Wuhan 430070, China; zym126135@126.com (Y.Z.); zhoujiahao@brunp.com.cn (J.Z.); dingwei@mails.swust.edu.cn (W.D.); y_zhang@whut.edu.cn (Y.Z.); 3 Hubei Key Laboratory of Mineral Resources Processing and Environment, Wuhan 430070, China; 4 State Environmental Protection Key Laboratory of Mineral Metallurgical Resources Utilization and Pollution Control, Wuhan University of Science and Technology, Wuhan 430081, China

**Keywords:** chemically supported anionic functional ionic liquid, polystyrene [1-butyl-3-methyl-imidazolium][nitrate], ultrasound assistance, vanadium, adsorption performance

## Abstract

In this study, the chemically supported ionic liquids (CSILs) were synthesized by ultrasound irradiation (UI) to improve the preparation process and further strengthen the adsorption performance of CSILs towards vanadium (V). The impacts of UI and conventional mechanic stirring (CMS) on the synthesis and adsorption characteristics of polystyrene [1-butyl-3-methylimidazolium][nitrate] (PS[C_4_mim][NO_3_]) were comparatively investigated. The experimental results demonstrate that ultrasound can dramatically shorten the preparation time from 1920 min to 15 min, and HNO_3_ dosage is reduced by 15.79%. Under the same adsorption conditions, the CSILs synthesized by UI achieve the maximal adsorption capacity towards vanadium (V) as 248.95 mg/g at 150 min, while the CSILs processed by CMS reach 223.90 mg/g at 105 min. Particularly, the adsorption capacity of CSILs synthesized by UI can be maintained as 96.42% of the initial value after 10 cycles of adsorption–desorption, while that of CSILs processed by CMS maintain as 94.87%. The adsorption isotherm and kinetics fitting demonstrate that vanadium (V) adsorption by two CSILs is dominated by chemisorption as a single molecular layer. Additionally, the adsorption reaction of vanadium (V) by these two CSILs are both endothermic, and entropy increases. Fourier transform infrared, scanning electron microscopy, and energy spectrometry analyses prove that PS[C_4_mim][NO_3_] is successfully prepared by UI and CMS methods, and ultrasound waves will not destroy the intact spherical structure of the support resins. The current work provides a novel insight for the efficient synthesis of CSILs, which is also a potential technique for improving the adsorption performance of the adsorbents towards valuable metals.

## 1. Introduction

Vanadium, as modern industrial ‘monosodium glutamate’, is broadly applied as a raw material in many areas, including metallurgy, new energy, national defense, aerospace, and so on [1,2]. Thus, many developed countries have included it in the strategic metal lists to ensure national security [3,4]. The reserves of vanadium resources in China rank first in the world, and hydrometallurgical leaching is indispensable for vanadium extraction from vanadium-containing resources, for instance vanadium-bearing shale, vanadium titanium magnetite, spent vanadium-based catalysts, fly ash, and petroleum coke. However, abundant impurities are generally dissolved with vanadium in the leachate [5], which seriously impedes the production of vanadium-containing compounds with high purity [6]. Herein, the efficient purification and enrichment of vanadium from complicated vanadium-containing leachate has been developed into a hotspot in the vanadium extraction area.

Ionic liquids (ILs), known as ‘green solvents’, have been adopted to separate and recover valuable components, due to their remarkable selectivity, designable structure, and environmental friendliness, etc. [7,8]. Recently, ILs have attracted more and more attention over vanadium separation from vanadium-containing solutions [9,10]. Nevertheless, ILs still cannot avoid the issues existing in the conventional liquid–liquid extraction process. Therefore, scholars have engaged to exploit solvent-impregnated resins (SIRs) [11,12], ILs-impregnated resins (IL-IRs) [13,14], and chemically supported ILs (CSILs) [15,16] so as to improve the extraction efficiency of extractants. According to our previous work, it was found that alkyl-based supported ILs can be designed into a variety of ILs with specific functions by changing the type or structure of cation and anion during the chemical grafting process, which exhibit a favorable affinity for vanadium [17,18]. However, the preparation process of CSILs are basically performed with auxiliary mechanic stirring in constant-temperature water bath oscillators [19,20] or magnetic stirrers [21]. The traditional synthesis processes commonly suffer from low preparation efficiency, and such-prepared CSILs display relatively poor adsorption performance towards targeted metals [22], which seriously hinder their industrial applications.

In recent years, ultrasound has been given extensive attention in various fields, including chemical synthesis, hydrometallurgy, and crystallization, attributable to its remarkable advantages of enhancing reaction rate, reducing energy consumption, and improving reaction yields, etc. [23,24,25]. Many scholars attempted to enhance the impregnation efficiency of specific components onto support with the assistance of ultrasonic waves, and such-prepared materials in turn exhibit excellent performance, including catalytic, adsorption performance, and so on [26,27,28]. Xiao et al. [26] investigated the adsorption performance of dibenzothiophene onto Ag/Cu/Fe-supported activated carbons (ACs) synthesized via ultrasound physical impregnation. The results demonstrate that dibenzothiophene adsorption capacity onto Ag/ACs and Cu/ACs prepared by ultrasound is obviously higher than that prepared by traditional impregnation, which is attributed to the fact that ultrasound induces the metallic particles to become finer and disperse more even. Tangestaninejad et al. [27] introduced vanadium polyoxometalate (PVMo) onto mesoporous molecular sieve MCM-41 by impregnation and adsorption methods under the action of ultrasound. It was reported that ultrasonic radiation significantly improved structural homogeneity, shortened reaction time, and increased product yields. Our group [28] adopted ultrasound irradiation to prepare SIRs and discovered that the impregnation efficiency was significantly enhanced compared with the conventional impregnation process. Furthermore, the adsorption properties of such-prepared SIRs are better compared to those synthesized by traditional methods, ascribable to the fact that the extractants disperse more uniformly into the deep pores of the resins with the action of ultrasonic wave. From the existing research, it was discovered that the application of ultrasound can bring many conducive effects to the preparation of novel functional materials. Ultrasound generally provides a unique physical and chemical environment, which allows difficult-to-achieve reactions to occur under routine conditions [29]. Nevertheless, the application of ultrasonic irradiation to synthesize CSILs and the adsorption performance of valuable metals onto such-prepared CSILs is rarely reported.

This study compared in detail the impacts of various preparation conditions on the synthesis process and adsorption performance of CSILs using conventional mechanic stirring (CMS-CSILs) and ultrasound irradiation (UI-CSILs) techniques. Furthermore, the adsorption mechanisms of these two CSILs were revealed through the investigation of adsorption isothermal data, kinetics, and thermodynamics. Thereafter, the cyclic adsorption stability of UI-CSILs and CMS-CSILs were explored and evaluated by multiple adsorption–desorption cycles. Finally, such-prepared CSILs were also characterized and analyzed by Fourier infrared spectrometer (FTIR) and scanning electron microscope furnished with energy-dispersive spectrometer (SEM–EDS) to probe the influence of ultrasound on loading characterizations of anionic functional group ionic liquids on the support. The present work aims to disclose the influence of ultrasonic wave on the synthesis and adsorption performance of CSILs and provide an efficient pathway for the synthesis of functional adsorbents.

## 2. Experiment and Characterization Methods

### 2.1. Raw Materials and Instruments

The 1-butyl-3-methylimidazolium chloride supported on Merrifield resins, PS[C_4_mim][Cl], was prepared according to our previous work, which is set as precursor for subsequent preparation of PS[C_4_mim][NO_3_] [15]. The Merrifield resins were purchased from Tianjin Nankai Hecheng Technology Co., Ltd., Tianjin, China; the Cl content, divinyl benzene crosslinking degree, and particle size is 3.7 mmol/g, 1%, and 0.074 mm–0.15 mm, respectively. Concentrated HNO_3_ and NaVO_3_ purchased from Shanghai Maclean Biochemical Co., Ltd., Shanghai, China are both of analytical grade. The stock vanadium (V) solution was prepared via dissolving NaVO_3_ in deionized water. All other chemical reagents adopted in this work are also of analytical grade.

The synthesis of CSILs was carried out in an ultrasonic chemical reaction system (VS-SHX-1500W, Wuxi Voshin Instrument Manufacturing Co., Ltd., Wuxi, China) combined with a thermostatic bath oscillator (SHA-2, Jiangsu Jintan Yitong Electronics Co., Ltd., Changzhou, China). The obtained samples were desiccated in a vacuum oven (Model DZF-6020, Shanghai Sopo Instruments Co., Ltd., Shanghai, China).

### 2.2. Synthesis of CSILs

**Figure 1:**
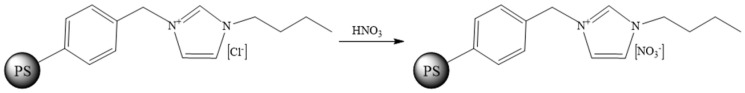
The conversion reaction from PS[C_4_mim][Cl] to PS[C_4_mim][NO_3_].

The preparation principle of PS[C_4_mim][NO_3_] by PS[C_4_mim][Cl] is presented in Figure 1. The synthesis of PS[C_4_mim][NO_3_] was contrastively conducted by conventional mechanic stirring (CMS) and ultrasound irradiation (UI) processes.

**Figure 2:**
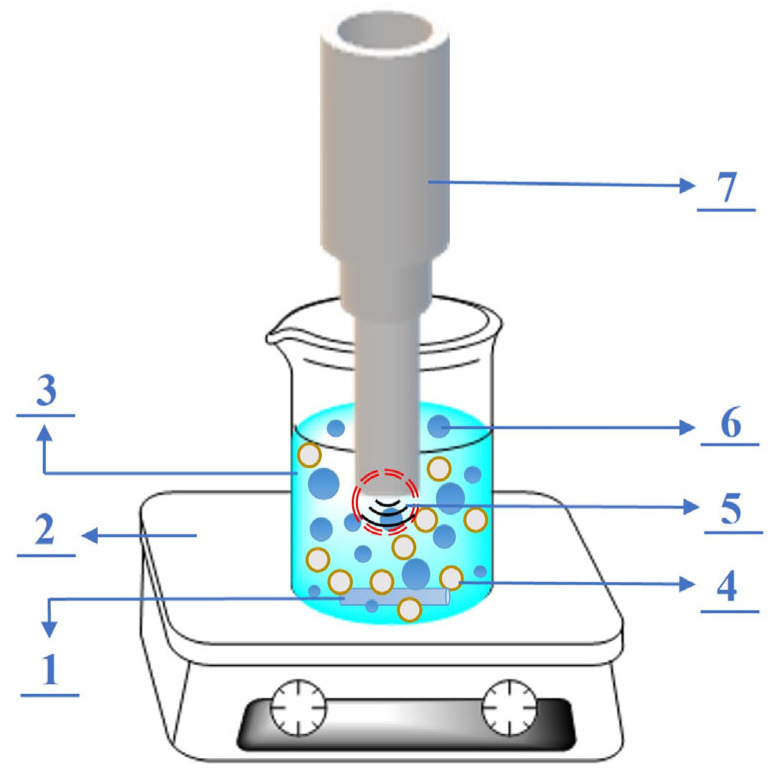
Ultrasound-assisted preparation process: 1—magnetic rotor, 2—magnetic agitator, 3—nitric acid solution, 4—PS[C_4_mim][Cl], 5—ultrasound cavitation phenomenon, 6—cavitation bubbles, 7—ultrasonic probe.

UI process (Figure 2): A certain amount of concentrated HNO_3_ was diluted with deionized water, which was blended with the dried PS[C_4_mim][Cl] at a liquid to solid ratio (L/S) of 150:1 mL/g under the pre-mechanic agitation. Subsequently, an ultrasound probe was inserted into the mixture, and the synthesis reaction was carried out at different ultrasonic power, reaction time, and nitric acid amounts. When the reaction was completed, the solid was filtrated using a Brinell funnel. Afterwards, the solid samples were washed several times by deionized water so as to remove the residual HNO_3_. Finally, the filtered solid samples were heated at 60 °C for 12 h in a vacuum oven to obtain dried UI-CSILs, that is PS[C_4_mim][NO_3_].

CMS process: The synthesis process of CMS-CSILs was parallel to that of UI-CSILs. The sole distinction is that the former was carried out in a thermostatic bath oscillator by mechanic stirring (120 rpm) without ultrasound.

The exchange rate of [NO_3_]^−^ and [Cl]^−^ was calculated by measuring the distinction in the concentration of NO_3_^−^ within the solution before and after the synthesis reaction. The ion exchange ratio (*E*) was calculated according to Equation (1).

(1) $$E=\frac{\left(N_{0}-N_{e}\right)}{N_{c}}\times 100\%$$

where *N*_0_ and *N*_e_ are the amount of substance of NO_3_^−^ in the solution before and after reaction (mol), respectively; *N*_c_ is the amount of substance of Cl^−^ on the precursor PS[C_4_mim][Cl] (mol).

### 2.3. Static Adsorption

The pH value of the vanadium (V) solutions was regulated to 1.6 and then blended with dried PS[C_4_mim][NO_3_] at L/S of 200:1 mL/g. Afterwards, the mixture in a conical flask was uniformly oscillated (120 rpm) at various contact times and adsorption temperatures in a thermostatic bath oscillator. Finally, the mixed solution was filtered to obtain the solid samples and filtrate. The equilibrium adsorption capacity, *Q*_e_ (mg/g), of CSILs towards vanadium (V) was calculated by Equation (2).

(2) $$Q_{e}=\frac{(C_{0}-C_{e})V}{m}$$

where *C*_e_ and *C*_0_ are the equilibrium and initial vanadium (V) concentrations in aqueous solutions (mg/L), respectively; *V* is the feed solutions’ volume (L); and *m* is the weight of the dried CSILs (g).

### 2.4. Cyclic Use

Ten cyclic adsorption–desorption experiments were carried out to evaluate the adsorption stability of UI-CSILs and CMS-CSILs. The adsorbed CSILs and 5 mol/L HNO_3_ solution were mixed at L/S of 400:1 mL/g in a conical flask and oscillated in a thermostatic bath oscillator for 5 h to finish the desorption process. The desorption ratio (*D*_V_) of vanadium (V) and the maintained ratio (*η*_n_,%) of vanadium (V) adsorption capacity at the nth cycle compared to the initial vanadium (V) adsorption capacity were calculated based on Equations (3) and (4), respectively. The desorption ratio of vanadium (V) from these two CSILs was kept higher than 97% each time.

(3) $$D_{V}=\frac{m_{\mathrm{ve}}}{m_{v0}}\times 100\%$$

(4) $$\eta_{n}=\frac{Q_{n}}{Q}\times 100\%$$

where *m*_v0_ is the mass of vanadium (V) adsorbed on PS[C_4_mim][NO_3_] (mg); *m*_ve_ is the weight of vanadium (V) in the solution after desorption (mg); *Q*_n_ is the adsorption capacity of vanadium (V) at the *n*th cycle (mg/g); and *Q* is the initial adsorption capacity of vanadium (V) (mg/g).

### 2.5. Characterization and Analysis Method

The vanadium (V) concentration in the solution was detected by ferrous ammonium sulfate titration method (GB/T 8704.5-2020). The N content in PS[C_4_mim][Cl] was measured via elemental analyzer (Vario EL cube, Elementar). Ion chromatography (Essentia IC-16, Shimadzu Global Laboratory Consumables Co., Ltd. Kyoto, Japan) was performed to determine the NO_3_^−^ concentration in the aqueous solution. Fourier transform infrared spectrometer (Nicolet™ iS™10, Thermo Nicolet Corporation Co., Ltd. Waltham, MA, USA) was applied to analyze the characteristic chemical bonds on PS[C_4_mim][Cl] and PS[C_4_mim][NO_3_] before and after adsorption. The ionic liquid films and element distribution on the resin surface of PS[C_4_mim][NO_3_] before and after adsorption were observed and analyzed by scanning electron microscope (JSM-IT300, JEOL, Tokyo, Japan) equipped with energy-dispersive spectrometer (X-Max 20, Oxford, Abingdon, UK). The SevenCompact^TM^ pH/ion meter (S220, Mettler-Toledo Instrument Co., Ltd. Columbus, Ohio, USA) was adopted to measure the pH value of the aqueous solution.

## 3. Results and Discussions

### 3.1. Effects of Preparation Parameters on the Properties of PS[C_4_mim][NO_3_]

#### 3.1.1. Ultrasonic Power

**Figure 3:**
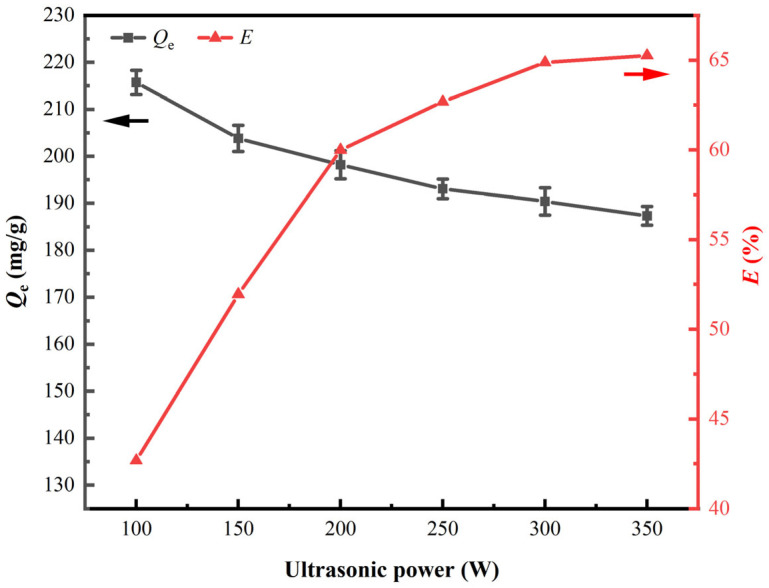
Impacts of ultrasound power on the synthesis and adsorption properties of PS[C_4_mim][NO_3_].

The amount of substance of HNO_3_ is 1.6 times that of the theoretical value (*N*_HNO3_ = 1.6), and reaction time is 15 min. The impacts of ultrasound power on the synthesis of CSILs and vanadium (V) adsorption capacity onto them were investigated under 100 W to 300 W. Corresponding results are displayed in Figure 3.

Figure 3 shows that the ion exchange ratio of [NO_3_]^−^ and [Cl]^−^ onto UI-CSILs first significantly augments with the elevation of ultrasound power from 100 W to 300 W, and then it almost achieves equilibrium when the ultrasound power is higher than 300 W. The results demonstrate that ultrasound radiation enhances the ion exchange reaction between HNO_3_ and PS[C_4_mim][Cl] via accelerating the dispersion of [NO_3_]^−^ in the pores of the resins and intensifying exchange efficiency between [NO_3_]^−^ and [Cl]− [30]. Acoustic cavitation, involving the nucleation, growth, and instantaneous bursting of cavitation bubbles [23], will provide sufficient energy for the synthesized reaction through transient cavitation bubbles and adiabatic collapse during the UI process.

Interestingly, as the [C_4_mim][NO_3_] content within the UI-CSILs elevates, vanadium adsorption capacity onto them decreases instead. As is known to us, the radius of [Cl]^−^ is much smaller than that of [NO_3_]^−^, and, hence, the van der Waals forces between [C_4_mim]^+^ and [Cl]^−^ onto PS[C_4_mim][Cl] is much weaker compared to that onto PS[C_4_mim][NO_3_] [31]. Van der Waals forces and hydrogen bonding are the dominant factors affecting ion interaction energy, where the presence of water and other solvents significantly impacts the activity and physical properties of ILs, such as polarity, viscosity, and conductivity [32]. Also, water affects the reaction rate and selectivity of ILs [33]. When the radius of the anion ions decreases, the higher the enthalpy of interaction with water, and the easier it dissolves in water. Therefore, [C_4_mim][Cl] has a larger enthalpy of interaction with water, and it is more soluble in water compared to [C_4_mim][NO_3_] [34]. At ultrasonic power of 100 W, the anions of the ILs loaded in UI-CSILs mainly exist in the form of [Cl]^−^; hence, vanadium (V) adsorption capacity is maximal. With the progressive increase in ultrasonic power, more binding sites between [C_4_mim]^+^ and [Cl]^−^ are gradually occupied by [NO_3_]^−^, in which it is relatively harder for the anions on UI-CSILs to exchange with vanadium anions in the aqueous solutions. Furthermore, the elevation of ultrasonic power increases the number of cavitation bubbles, high-speed microjets, and microbeam flows, which allows more ILs to enter the deep pores of the resins. However, the supported ILs in the deeper pores are difficult to react with vanadium (V) at a relatively short contact time of 1.5 h, leading to an increasingly low vanadium (V) adsorption capacity by UI-CSILs. Herein, in order to obtain a relatively high ion exchange ratio and adsorption capacity, the optimum ultrasound power is selected as 200 W for subsequent synthesis of PS[C_4_mim][NO_3_] by UI.

#### 3.1.2. Reaction Time

**Figure 4:**
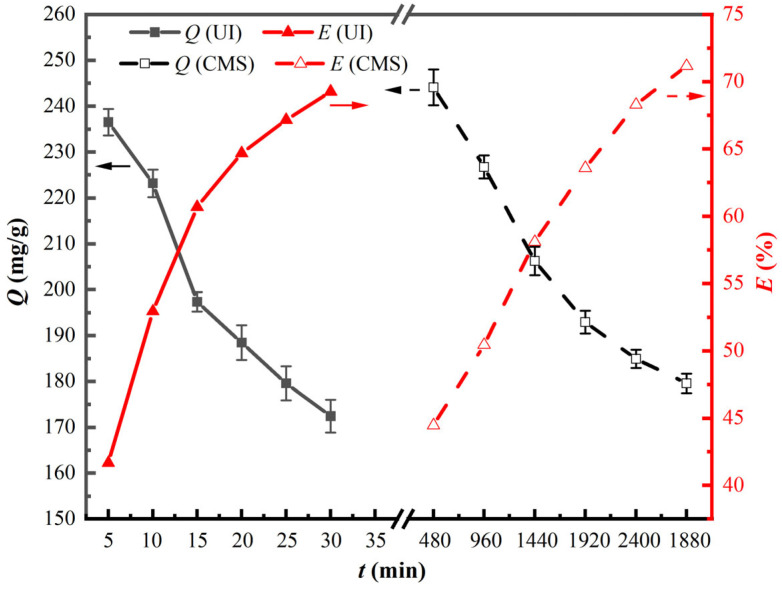
Impacts of reaction time on the synthesis and adsorption properties of PS[C_4_mim][NO_3_].

The impacts of reaction time on the synthesis of UI-CSILs and adsorption capacity towards vanadium (V) were investigated from 5 min to 30 min. As a comparison, the synthesis of CMS-CSILs was conducted at various reaction times. Corresponding results are displayed in Figure 4.

Figure 4 demonstrates that the ion exchange ratios of UI-CSILs and CMS-CSILs both first markedly ascend with the extension of reaction time, and then the increase trend slows down. The ion exchange ratio and vanadium (V) adsorption capacity onto UI-CSILs at 15 min reach 60.7% and 197.36 mg/g, respectively, while those of CMS-CSILs at 1920 min achieve 63.58% and 192.91 mg/g, severally. The results indicate that ultrasound radiation not only dramatically shortens synthesis time of PS[C_4_mim][NO_3_], but also slightly improves the adsorption capacity towards vanadium (V). Hence, ultrasound-assisted preparation of PS[C_4_mim][NO_3_] is accepted as more efficient compared to conventional methods [35]. Of course, the experimental results contradict the common understanding that the larger the proportion of NO_3_^−^ on CSILs, the higher the adsorption capacity towards vanadium (V). This may be ascribed to the high viscosity of the supported ILs and slow diffusive motion of IL molecules during the CMS process. It is speculated that ILs are preferentially loaded onto the resins by stacking on the pores’ wall and forming a multimolecular layer. When ultrasound is introduced into the preparation process, ultrasonic cavitation would produce high-speed microjets, generating high-pressure shock waves that continuously impact the resin pores and ILs. Hence, the diffusion of ILs within the resins’ pore channels is promoted, which induces the ILs to uniformly spread on the pore surface as a single molecular layer [36]. This makes more active ILs loaded onto UI-CSILs react with vanadium (V), resulting in higher vanadium (V) adsorption capacity in comparison to CMS–CSILs. Therefore, the optimal sonication time for the UI process is determined as 15 min, considering the economic effectiveness and relatively high vanadium (V) adsorption capacity.

#### 3.1.3. HNO_3_ Dosage

**Figure 5:**
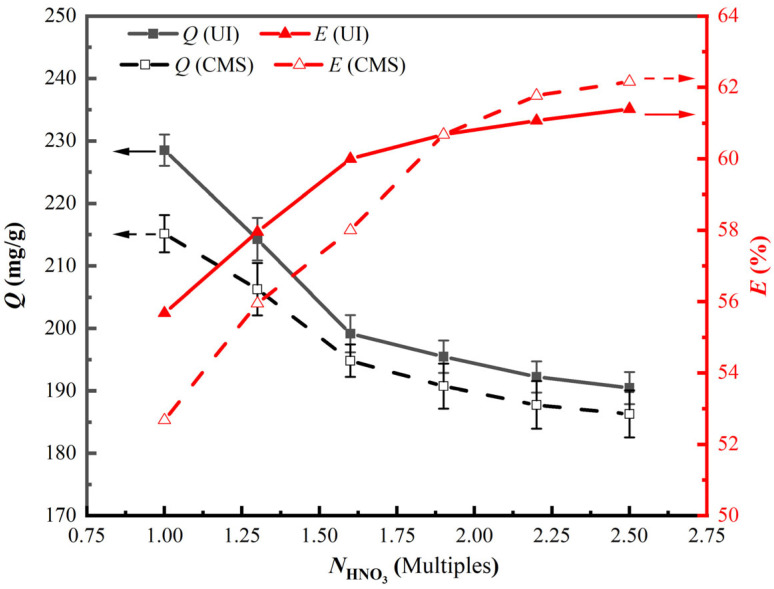
Impacts of HNO_3_ dosage on the preparation and adsorption properties of PS[C_4_mim][NO_3_].

The impacts of HNO_3_ dosage on the ion exchange ratios and vanadium adsorption capacity onto PS[C_4_mim][NO_3_] were investigated at *N*_HNO3_ of 1.0, 1.3, 1.6, 1.9, 2.2, and 2.5 times that of the theoretical amount. Corresponding results are displayed in Figure 5.

As is presented in Figure 5, the ion exchange ratios of UI-CSILs and CMS-CSILs both firstly augment with the rise in the amount of HNO_3_, and then it tends to reach equilibrium with further elevated HNO_3_ dosage. At *N*_HNO3_ of 1.6, the ion exchange ratio of UI-CSILs is approaching equilibrium, while the equilibrium point of CMS-CSILs is 1.9. When *N*_HNO3_ is of 1.0 to 1.9, ILs are dominantly filled into resins’ pores as a multimolecular layer during the CMS process. In contrast, ILs are loaded onto the resins’ pore channel as a monomolecular layer by spreading on the pore surface with the assistance of ultrasound irradiation. Hence, the ion exchange ratio of UI-CSILs is significantly improved compared to that of CMS-CSILs at *N*_HNO3_ range of 1.0 to 1.9. In particular, vanadium (V) adsorption capacity onto UI-CSILs is always higher than that onto CMS-CSILs at the studied HNO_3_ dosage.

When *N*_HNO3_ is higher than 1.9, it is distinct that the ion exchange ratios of UI-CSILs are always lower than that of CMS-CSILs. It is speculated that ultrasonic cavitation would induce a break of chemical bonds between the ILs and the resins’ pore surface, leading to the desorption of some ILs loosely loaded on the resins [37]. The vanadium (V) adsorption capacities onto UI-CSILs and CMS-CSILs- both follow the principle that the larger the proportion of NO_3_^−^ within the ILs is, the lower the adsorption capacity towards vanadium (V). Therefore, the optimal amount of HNO_3_ is selected as *N*_HNO3_ of 1.6 and 1.9 for UI-CSILs and CMS-CSILs, respectively.

### 3.2. Static Adsorption Performance of PS[C_4_mim][NO_3_]

The elemental analysis of PS[C_4_mim][NO_3_] synthesized by two methods is presented in Table 1. It is obvious that the N content of UI-CSILs is slightly lower than that of CMS-CSILs, which is consistent with the experimental results. The deviation between the theoretical values and element analysis data of C, H, and N contents on PS[C_4_mim][NO_3_] synthesized by two methods is presented in Tables S1 and S2, respectively. The adsorption performance of UI-CSILs and CMS-CSILs prepared at optimal synthesis parameters towards vanadium (V) were comparatively investigated under various adsorption conditions.

#### 3.2.1. Impacts of Contact Time

**Figure 6:**
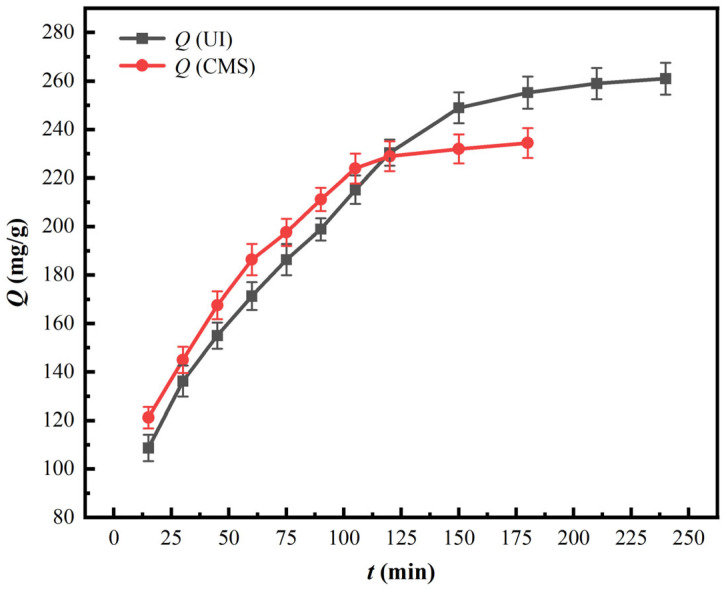
Impacts of contact time on the adsorption properties of PS[C_4_mim][NO_3_].

To investigate the impacts of contact time over the adsorption properties of PS[C_4_mim][NO_3_], corresponding experiments were conducted under an initial pH value of 1.6, vanadium (V) concentration of 2500 mg/L, and adsorption temperature of 25 °C. Corresponding results are displayed in Figure 6.

As is presented in Figure 6, vanadium (V) adsorption capacity onto UI-CSILs and CMS-CSILs both first rapidly augment with the extension of contact time, and then the growth trend slows to equilibrium. Vanadium (V) adsorption capacity onto CMS-CSILs attains equilibrium at 105 min as 223.90 mg/g, while the adsorption capacity of UI-CSILs continuously elevates after 105 min and finally achieves equilibrium at 150 min as 248.95 mg/g. Nevertheless, vanadium (V) adsorption capacity onto the former is always slightly higher than that onto the latter before 120 min, which is ascribed to the disparate existence forms of ILs on the resins. Commonly, metal ions adsorption on adsorbents consists of three steps: (1) diffusion of metal ions through the liquid films on the resin surface; (2) intraparticle diffusion of metal ions in the resin pores; and (3) the reaction between metal ions and the functional groups. Generally, the intra-particle diffusion process is accepted as the rate-controlling step [38]. As is stated in Section 3.1.1, the ILs loaded on UI-CSILs enter the deeper pore channels compared to that on CMS-CSILs. Hence, it takes more time for vanadium ions to diffuse into the deep pore channels of the resin and react with the loaded ILs, resulting in the longer adsorption equilibrium time of UI-CSILs. In contrast, as the reaction time extends to longer than 120 min, vanadium (V) adsorption capacity onto UI-CSILs is apparently higher than that of CMS-CSILs, ascribed to the fact that the ILs in deeper pores can finally react with the vanadium ions.

#### 3.2.2. Impacts of Initial Vanadium (V) Concentration

**Figure 7:**
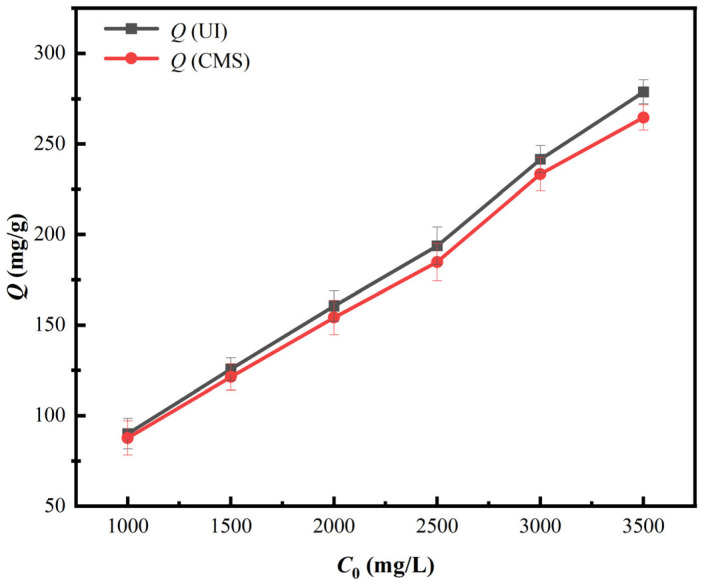
Impacts of initial concentration on the adsorption properties of PS[C_4_mim][NO_3_].

To investigate the impacts of initial vanadium (V) concentration over the adsorption properties of PS[C_4_mim][NO_3_], corresponding experiments were conducted under initial pH value of 1.6, contact time of 150 min for UI-CSILs (105 min for CMS-CSILs), and adsorption temperature of 25 °C. Corresponding results are displayed in Figure 7.

As is presented in Figure 7, vanadium (V) adsorption capacity onto UI-CSILs and CMS-CSILs augments linearly with the elevation in initial vanadium concentration. As initial vanadium concentration increases, a larger differential concentration is formed between aqueous solutions and PS[C_4_mim][NO_3_]. This promotes the diffusion of the vanadium into resin pores and improves the possibility of anion exchange between the vanadium anions and the loaded ILs, which, in turn, leads to a remarkable increase in vanadium (V) adsorption capacity onto these two CSILs. Furthermore, vanadium (V) adsorption capacity onto UI-CSILs is always higher than that of CMS-CSILs at the same initial vanadium concentration, and the trend becomes more obvious with the elevation in initial vanadium concentration. As is stated above, the ultrasound would induce the existence form of ILs to transform from a multimolecular layer to a single molecular layer. As the vanadium concentration in the solution augments, the ILs uniformly supported on the resin pores as a single molecular layer can efficiently react with vanadium anions in comparison to that loaded as multimolecular layers.

#### 3.2.3. Impacts of Adsorption Temperature

**Figure 8:**
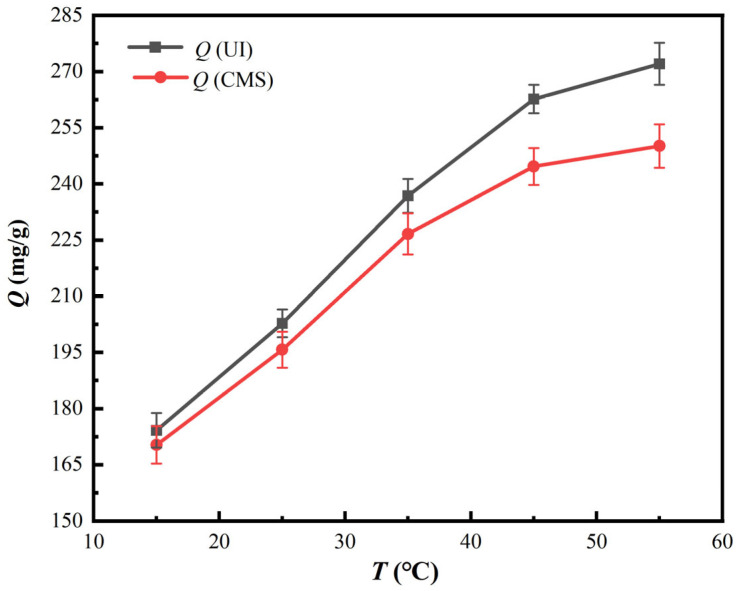
Impacts of adsorption temperature on the adsorption properties of PS[C_4_mim][NO_3_].

To probe the impacts of adsorption temperature on vanadium (V) adsorption capacity onto PS[C_4_mim][NO_3_], corresponding experiments were conducted under initial pH value of 1.6, contact time of 150 min for UI-CSILs (105 min for CMS-CSILs) and initial vanadium concentration of 2500 mg/L. The results are displayed in Figure 8.

As is presented in Figure 8, vanadium (V) adsorption capacity onto UI-CSILs augments from 174.19 mg/g to 272.05 mg/g as the adsorption temperature elevates from 15 °C to 55 °C, while that onto CMS-CSILs increases from 170.27 mg/g to 250.13 mg/g. It is apparent that vanadium (V) adsorption capacity first augments linearly with the elevated temperature, and then the increase trend slows to equilibrium with further increase. The elevation of adsorption temperature would accelerate the migration and diffusion process of vanadium anions from the aqueous solution to the PS[C_4_mim][NO_3_] [22], which promote the reaction between vanadium anions with the loaded ILs. Nevertheless, the active adsorption sites of the loaded ILs on the resins are limited, and are gradually occupied by vanadium anions. Hence, vanadium (V) adsorption capacity is approaching to saturation with the further elevation in adsorption temperature. Furthermore, the difference among vanadium (V) adsorption capacity onto UI-CSILs and CMS-CSILs is observably elevated with the increasing adsorption temperature. As is stated in above sections, the ILs existing as a single molecular layer can react with vanadium anions more efficiently in comparison to a multimolecular layer, which is further augmented with the ascending temperature.

### 3.3. Adsorption Mechanisms of PS[C_4_mim][NO_3_] Towards Vanadium (V)

#### 3.3.1. Adsorption Isotherm of Vanadium(V) onto PS[C_4_mim][NO_3_]

**Figure 9:**
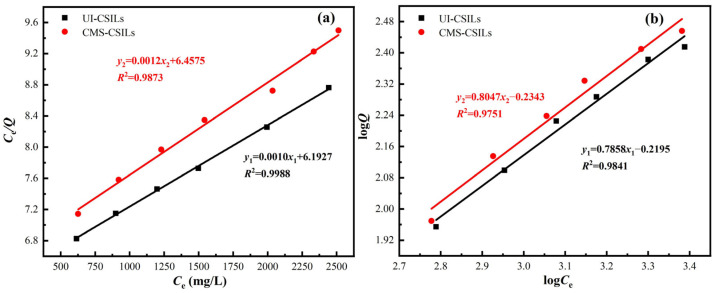
Fitting of vanadium (V) adsorption process by UI-CSILs and CMS-CSILs using Langmuir (**a**) and Freundlich isothermal models (**b**).

The Langmuir (Equation (5)) and Freundlich (Equation (6)) isotherm models are usually adopted to describe the adsorption equilibrium in liquid–solid systems [39]. The Langmuir model generally assumes that the adsorption reaction of metal ions takes place on a homogeneous surface as a single molecular layer, in which there is no interaction among the adsorbed ions. The Freundlich model hypothesizes that the adsorption reaction of metal ions takes place on a heterogeneous surface as multimolecular layers. The Langmuir and Freundlich models were adopted to analyze the adsorption experimental data of UI-CSILs and CMS-CSILs in Figure 6, and corresponding fitting results and relevant fitting parameters are displayed in Figure 9 and Table 2, respectively.

(5) $$\frac{C_{e}}{Q}=\frac{1}{Q_{0}K_{L}}+\frac{C_{e}}{Q_{0}}$$

(6) $$\log Q=\log K_{F}+\frac{1}{n}\log C_{e}$$

where *Q* is the equilibrium vanadium (V) adsorption capacity onto PS[C_4_mim][NO_3_] (mg/g); *C*_e_ is the equilibrium vanadium (V) concentration in aqueous solution (mg/L); *Q*_0_ is the theoretical maximal vanadium (V) adsorption capacity onto PS[C_4_mim][NO_3_] (mg/g); *K*_L_ is the Langmuir equilibrium adsorption constant (L/g); *K*_F_ is the Freundlich adsorption constant, referring to the adsorption capacity of PS[C_4_mim][NO_3_] (L/g); *n* is the Freundlich isothermal adsorption correlation coefficient, reflecting the inhomogeneity of PS[C_4_mim][NO_3_] and the strength of the adsorption reaction of vanadium (V).

As is presented in Figure 9, the goodness of fit (*R*^2^) of the Langmuir model is closer to 1 compared to that of the Freundlich mode for both UI-CSILs and CMS-CSILs. This indicates that vanadium (V) adsorption onto these two CSILs is well depicted by the Langmuir adsorption model, where vanadium (V) is adsorbed as a single molecular layer on PS[C_4_mim][NO_3_]. Table 2 demonstrates that the theoretical maximal vanadium (V) adsorption capacity onto UI-CSILs is clearly higher than that on CMS-CSILs ascribable to the fact that there are more active ILs within UI-CSILs existing as a single molecular layer. This further corroborates with the speculation in Section 3.1.1 and Section 3.1.2 concerning the different existence form of ILs on UI-CSILs and CMS-CSILs.

#### 3.3.2. Adsorption Kinetics of Vanadium (V) onto PS[C_4_mim][NO_3_]

**Figure 10:**
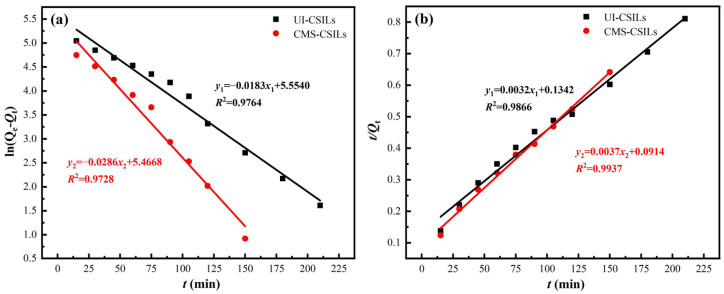
Fitting of vanadium (V) adsorption kinetic process by UI-CSILs and CMS-CSILs using pseudo-first-order (**a**) and pseudo-second-order adsorption model (**b**).

The adsorption kinetics reflects the adsorption process of metal ions by the adsorbents, which is significant for the evaluation of the rate-controlling step during the entire adsorption process. The experimental data in Figure 7 were depicted with the pseudo-first-order kinetic model [40] (Equation (7)) and the pseudo-second-order kinetic model [41] (Equation (8)) to investigate the adsorption mechanism of vanadium (V) on UI-CSILs and CMS-CSILs. Corresponding fitting results are displayed in Figure 10, and relevant fitting parameters are exhibited in Table 3.

(7) $$\ln(Q_{e}-Q_{t})=\ln Q_{e}-k_{1}t$$

(8) $$\frac{t}{Q_{t}}=\frac{1}{k_{2}{Q_{e}}^{2}}+\frac{t}{Q_{e}}$$

where *Q*_e_ and *Q*_t_ represent the adsorption capacity of vanadium (V) onto PS[C_4_mim][NO_3_] (mg/g) at equilibrium and contact *t*, respectively; *k*_2_ and *k*_1_ are the pseudo-second-order and pseudo-first-order adsorption kinetic constants, respectively; *t* is the reaction time (min).

As is presented in Figure 10, the adsorption kinetic processes of vanadium (V) onto UI-CSILs and CMS-CSILs can be well depicted by the pseudo-second-order model, attributable to the fact that the *R*^2^ of it is relatively higher compared to that of the pseudo-first-order model. This demonstrates that the vanadium (V) adsorption process onto these two CSILs is dominated by chemisorption [42], where a chemical force is generated by electron transfer or sharing between PS[C_4_mim][NO_3_] and vanadium anions [43]. In addition, the adsorption kinetic constant (*k*_2_) of UI-CSILs is relatively lower than that of CMS-CSILs, leading to a long duration for the former to reach adsorption equilibrium, which is consistent with the phenomenon in Figure 6.

#### 3.3.3. Adsorption Thermodynamics of Vanadium (V) onto PS[C_4_mim][NO_3_]

**Figure 11:**
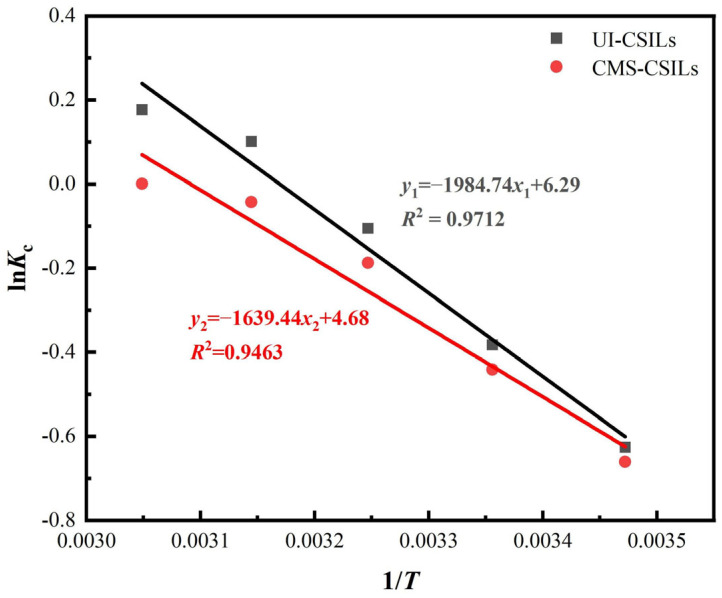
Fitting of adsorption thermodynamic of vanadium(V) by UI-CSILs and CMS-CSILs.

The adsorption temperature has a great influence on the adsorption properties of PS[C_4_mim][NO_3_]l; hence, the exploration of adsorption thermodynamics is vital for the revelation of the adsorption mechanism of vanadium (V) onto CSILs. According to the experimental data in Figure 8, the adsorption thermodynamic process of vanadium (V) is depicted by Equations (9) and (10) with ln*k*_c_ against 1/*T*. The enthalpy change and entropy change are calculated according to Equation (10) based on the slope and intercept in Figure 11. In addition, Gibbs free energy change is computed by Equation (11).

(9) $$K_{c}=\frac{C_{\mathrm{Ae}}}{C_{e}}$$

(10) $$\ln K_{c}=-\frac{\Delta H}{RT}+\frac{\Delta S}{R}$$

(11) ΔG=ΔH−TΔS

where *K*_c_ is the concentration equilibrium constant; *C*_Ae_ and *C*_e_ are the concentration of vanadium (V) on PS[C_4_mim][NO_3_] (mg/g) and in aqueous solution after adsorption (mg/L), respectively; R is the gas constant, 8.314 J/(mol·K); *T* is the adsorption Kelvin temperature (K); Δ*H*, Δ*S*, and Δ*G* are the enthalpy change (kJ/mol), entropy change (kJ/K/mol), and Gibbs free energy change (kJ/mol) of vanadium (V) adsorption reaction, respectively.

As is observed from Table 4, the enthalpy changes of vanadium (V) adsorption by UI-CSILs and CMS-CSILs are both positive, indicating that the adsorption processes of these two CSILs towards vanadium (V) are endothermic, and increased temperature is favorable in vanadium (V) adsorption. Meanwhile, the entropy changes of vanadium (V) adsorption by UI-CSILs and CMS-CSILs are greater than 0, implying that the disorder of the adsorbent surface increases with the reaction during vanadium (V) adsorption. In particular, the Gibbs free energy variation of UI-CSILs is always lower than that of the CMS-CSILs at the same adsorption temperature, which demonstrates that vanadium (V) adsorption reaction on the former is more likely to occur in comparison to that on the latter [44].

### 3.4. Cyclic Adsorption Stability

**Figure 12:**
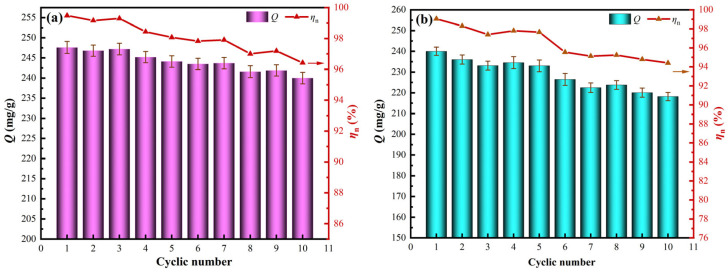
Cyclic adsorption stability of UI-CSILs (**a**) and CMS-CSILs (**b**).

The adsorption stability of UI-CSILs and CMS-CSILs is comparatively investigated during multiple cyclic use processes, and the corresponding results are displayed in Figure 12.

As is presented in Figure 12, vanadium (V) adsorption capacity onto UI-CSILs and CMS-CSILs both decrease with the augmentation of the cyclic number. It is evident that the decreasing trend for the adsorption capacity of CMS-CSILs is more evident than that of UI-CSILs. After the 10th cycle, vanadium (V) adsorption capacity onto UI-CSILs is 240.15 mg/g, which is equivalent to 96.42% of the initial value. Nevertheless, vanadium (V) adsorption capacity onto CMS-CSILs is only 218.66 mg/g, which is equal to 94.87% of the initial value. The results indicate that ILs loaded on the former are more stable than that on the latter, which is more difficult to lose into the aqueous solution during multiple cyclic uses. This also verifies that the ILs are uniformly distributed into the deeper pores of the UI-CSILs [45], which makes UI-CSILs maintain a relatively high adsorption stability.

### 3.5. Characterization and Analysis of PS[C_4_mim][NO_3_]

#### 3.5.1. Functional Groups Analysis

**Figure 13:**
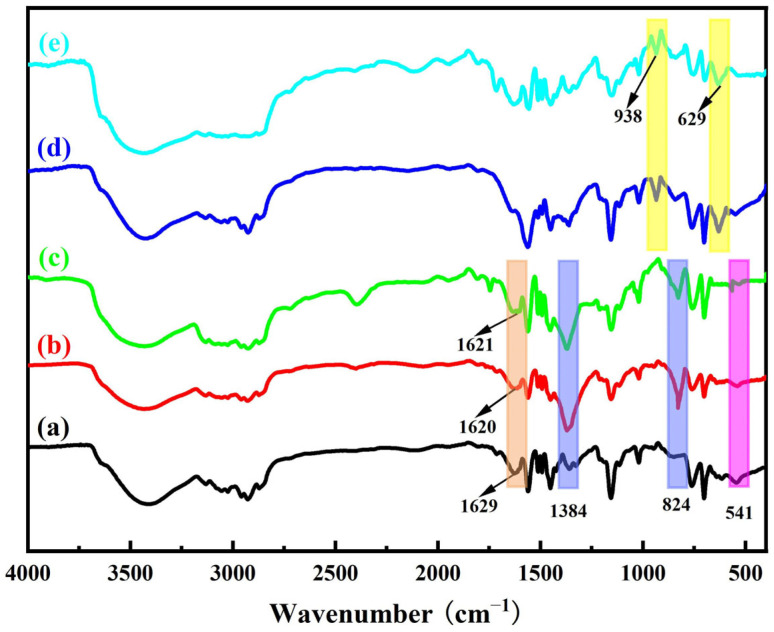
Functional groups of PS[C_4_mim][Cl] (a), UI-CSILs before (b) and after adsorption (d), CMS-CSILs before (c) and after adsorption (e).

The functional groups of PS[C_4_mim][Cl] and PS[C_4_mim][NO_3_] are characterized and analyzed by FTIR, and corresponding results are displayed in Figure 13.

As is presented in Figure 13, the peak located at 541 cm^−1^ in Figure 13a is assigned to [Cl]^−^, which weakens but does not disappear in Figure 13b,c. This implies that most of the [Cl]^−^ is exchanged during the grafting reaction process, but some remains unexchanged. The peaks at 824 cm^−1^ and 1384 cm^−1^ in Figure 13b,c are the symmetric and antisymmetric vibrational absorption peaks of [NO_3_]^−^, further indicating that [Cl]^−^ is successfully replaced by [NO_3_]^−^ by ion exchange. The alkyl absorption peak of 1629 cm^−1^ on PS[C_4_mim][Cl] shifts to 1621 cm^−1^ and 1620 cm^−1^ on PS[C_4_mim][NO_3_], which is ascribed to the interaction of the ILs cation and [NO_3_]^−^. The aforementioned results prove that both UI and CMS can successfully synthesize PS[C_4_mim][NO_3_] by chemical grafting processes. In Figure 13d,e, the peaks at 938 cm^−1^ and 629 cm^−1^ are assigned to the stretching vibration V=O and V-O bond [46,47], respectively, which indicates that vanadium (V) has already been adsorbed on PS[C_4_mim][NO_3_]. Particularly, the intensity of V=O and V-O bond on UI-CSILs is obviously higher than that on CMS-CSILs-, proving relative higher adsorption capacity of the former towards vanadium (V).

#### 3.5.2. Microstructure and Element Distribution Analysis

**Figure 14:**
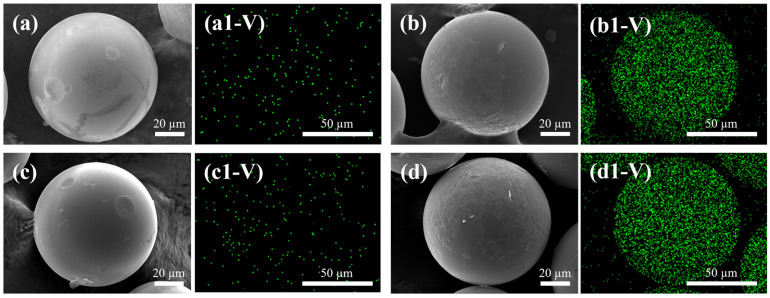
Microstructure and vanadium distribution of UI-CSILs before (**a**) and after adsorption (**b**), CMS-CSILs before (**c**) and after adsorption (**d**).

SEM–EDS is adopted to observe the microstructure changes and vanadium distribution onto UI-CSILs and CMS-CSILs before and after vanadium (V) adsorption, and the corresponding results are displayed in Figure 14.

As is presented in Figure 14a,c, both UI-CSILs and CMS-CSILs maintain an intact spherical structure, which demonstrates that the introduction of ultrasound waves will not destroy the skeleton of the resin particles. In Figure 14(b1,d1), it is visibly observed that vanadium (V) uniformly distributes on the surface of UI-CSILs and CMS-CSILs after adsorption, proving the high affinity of such-prepared adsorbents towards vanadium (V).

### 3.6. Comparisons of Vanadium Adsorption Performance of PS[C_4_mim][NO_3_] and Other Materials

As is presented in Table 5, the vanadium adsorption capacity of the PS[C_4_mim][NO_3_] prepared by UI is higher than the reported other materials, including the synthesized and commercial adsorbents. Furthermore, the adsorption selectivity and cyclic adsorption stability of CSILs are better than the common adsorbents based on the previous work of our group. This indicates that CSILs are an ideal vanadium adsorption material, which is expected to achieve efficient separation of vanadium from complicated vanadium-containing solutions. However, the cost of the current supporter for the CSILs, Merrifield resins, is more expensive than other reported carriers. Hence, it is urgent to find a substitute for Merrifield reins to promote the industrial application of CSILs in the future.

## 4. Conclusions and Prospect

In this work, the impacts of ultrasound irradiation (UI) and conventional mechanic stirring (CMS) on the preparation of chemically supported ionic liquids (CSILs) and the adsorption performance and mechanism of such-prepared PS[C_4_mim][NO_3_] towards vanadium (V) are comparatively investigated. The key findings are concluded as follows:

(1) The optimal preparation conditions for UI-CSILs are ultrasonic power of 200 W, reaction time of 15 min, and *N*_HNO3_ of 1.6. In comparison to the CMS method, the introduction of ultrasound obviously shortens the preparation time by 99.22%, and the HNO_3_ dosage can be reduced by 15.79%.

(2) Under the adsorption conditions of initial pH value of 1.6, liquid to solid ratio of 200:1 mL/g, adsorption temperature of 25 °C, and initial vanadium (V) concentration of 2500 mg/L, UI-CSILs achieve the maximal vanadium (V) adsorption capacity of 248.95 mg/g at 150 min, while CMS-CSILs reach 223.90 mg/g at 105 min. After the 10 cyclic uses, the adsorption capacity of vanadium (V) onto UI-CSILs remains at 96.42% of the initial value, while that onto CMS-CSILs remains at 94.87%. This demonstrates that the adsorption equilibrium time of UI-CSILs is slight longer than that of CMS-CSILs, but the adsorption stability of the former is obviously stronger than that of the latter.

(3) Both the adsorption of vanadium (V) onto UI-CSILs and CMS-CSILs are well depicted by the Langmuir adsorption isotherm model, demonstrating that vanadium (V) is adsorbed on PS[C_4_mim][NO_3_] as a single molecular layer. The adsorption kinetic processes of vanadium (V) by UI-CSILs and CMS-CSILs conform to the pseudo-second-order model, implying that the adsorption of vanadium (V) onto these two CSILs is dominated by chemisorption. Moreover, the adsorption reaction of vanadium (V) by UI-CSILs and CMS-CSILs are both endothermic, the entropy increases, and vanadium (V) adsorption on the former is more likely to occur compared to that on the latter.

(4) FTIR analysis proves the successful preparation of PS[C_4_mim][NO_3_] by UI and CMS methods, and both CSILs exhibit good affinity towards V (V). Furthermore, SEM–EDS analysis demonstrates that ultrasound waves will not damage the intact spherical structure of the support resins, which provide a basis for multiple cyclical use.

In the future, the work should be focused on revealing the deep mechanism through which UI improves the synthesis process and adsorption performance of CSILs, such as the distribution of flow field in the UI and CMS systems. In the meantime, the association between the anions and cations of the ILs, and the interaction of vanadium and impurities with ILs from the molecular and atomic level must continue to be studied.

## Figures and Tables

**Table 1:** The elemental analysis of PS[C_4_mim][NO_3_] synthesized by two methods.

Samples	N	C	H	O
UI-CSILs	7.04	69.73	8.32	7.92
CMS-CSILs	7.71	69.08	7.84	8.38

**Table 2:** Isothermal fitting parameters of vanadium (V) adsorption onto UI-CSILs and CMS-CSILs.

Supported ILs	Langmuir			Freundlich		
	*Q*_0_ (mg/g)	*K*_L_ (L/g)	*R* ^2^	*n*	*K*_F_ (L/g)	*R* ^2^
UI-CSILs	1000	1.6 × 10^−4^	0.9988	1.27	0.6033	0.9841
CMS-CSILs	833.33	1.9 × 10^−4^	0.9873	1.24	0.5830	0.9751

**Table 3:** Kinetic fitting parameters of vanadium (V) adsorption onto UI-CSILs and CMS-CSILs.

Supported ILs	Pseudo-First-Order			Pseudo-Second-Order		
	*Q*_e_	*k*_1_	*R* ^2^	*Q*_e_	*k*_2_	*R* ^2^
UI-CSILs	254.68	0.0183	0.9764	312.50	7.6 × 10^−5^	0.9866
CMS-CSILs	236.70	0.0286	0.9728	270.27	1.5 × 10^−4^	0.9937

**Table 4:** Thermodynamic fitting parameters of vanadium (V) adsorption by UI-CSILs and CMS-CSILs.

Supported ILs	*T* (K)	Δ*G* (kJ/mol)	Δ*S* (kJ/mol·K)	Δ*H* (kJ/mol)	*R* ^2^
UI-CSILs	288	1.44	0.0523	16.50	0.9712
	298	0.91			
	308	0.39			
	318	−0.13			
	328	−0.65			
CMS-CSILs	288	1.48	0.0422	13.63	0.9463
	298	1.05			
	308	0.63			
	318	0.21			
	328	−0.21			

**Table 5:** Comparisons of the adsorption performance of vanadium by some synthesized and commercial materials.

Number	Materials	Vanadium Adsorption Capacity, mg/g	Reference
1	Nickel–aluminum complex hydroxides	177.5	[48]
2	Modified activated carbon derived from natural hydroxyapatite	19.45	[49]
3	Nano-hydrous zirconium oxide-modified anion exchange resin	118.1	[3]
4	Titanium-based microspheres	153.2	[9]
5	Amino-functional polymeric	75.5	[45]
6	Ti-doped chitosan bead	210	[50]
7	Ion exchange resins TP220 and M4195	247.81 and 208.79	[40]
8	Anion exchange resin Dex-V	25.42	[47]
9	Anion exchange resins Amberlite IRA-400	27.4	[5]
10	Ion exchange resins D851 and D201	162 and 104	[7]
11	PS[C_4_mim][NO_3_] prepared by UI	248.95	This work

## Data Availability

The raw data supporting the conclusions of this article will be made available by the authors on request.

## Supplementary Materials

The following supporting information can be downloaded at: https://www.mdpi.com/article/10.3390/ma18061330/s1, Table S1: The deviation between the theoretical values (TE) and element analysis data (EAD) of C, H and N contents on PS[C_4_mim][NO_3_] synthesized by UI; Table S2: The deviation between the TE and EAD of C, H and N contents on PS[C_4_mim][NO_3_] synthesized by CMS.

## Abbreviations

CSILs	Chemically supported ionic liquids
UI	Ultrasound irradiation
CMS	Conventional mechanic stirring
PS[C_4_mim][NO_3_]	Polystyrene [1-butyl-3-methylimidazolium][nitrate]
UI-CSILs	Chemically supported ionic liquids synthesized by ultrasound irradiation
CMS-CSILs	Chemically supported ionic liquids synthesized by conventional mechanic stirring
ILs	Ionic liquids
SIRs	Solvent-impregnated resins
IL-IRs	Ionic liquids-impregnated resins
ACs	Activated carbons
PVMo	Vanadium polyoxometalate
FTIR	Fourier infrared spectrometer
SEM-EDS	Scanning electron microscope furnished with energy-dispersive spectrometer
PS[C_4_mim][Cl]	1-butyl-3-methylimidazolium chloride supported on Merrifield resins
