# Supplementary material for: Efficient Ultrasound-Assisted Synthesis of Chemically Supported Anionic Functional Group Ionic Liquids and Its Enhanced Adsorption Performance Towards Vanadium (V)
Source: Materials (Basel). 2025 Mar 18;18(6):1330. doi: 10.3390/ma18061330 (PMC11943831; doi:10.3390/ma18061330)
Supplement: Supplementary file 1 [file materials-18-01330-s001.zip › materials-3497590-supplementary.pdf]

Table S1. The deviation between the theoretical values (TE) and element analysis data (EAD) of C, H and N contents on PS[C_4mim][NO_3] synthesized by UI.

CSILs-UI	TE	EAD	Deviation
C	69.86	69.73	-0.19%
H	8.88	8.32	-6.31%
N	8.36	7.04	-15.79%

Table S2. The deviation between the TE and EAD of C, H and N contents on PS[C_4mim][NO_3] synthesized by CMS.

CSILs-CMS	TE	EAD	Deviation
C	69.82	69.08	-1.06%
H	8.87	7.84	-11.61%
N	8.38	7.71	-8.00%

It is worth noting that the N contents deviation for CSILs-UI and CSILs-CMS is -15.79% and -8.00%, respectively. In addition, the H contents deviation for CSILs-UI and CSILs-CMS is -6.13% and -11.61%, respectively. This is because that some HNO_3 physically adsorbed on the carrier resins were washed off during the process, which leads to the actual values are slightly lower than the theoretical values. Lastly, the deviation for C contents for CSILs-UI and CSILs-CMS is -0.19% and -1.06%, severally, which is obviously lower than that of N and H element. This is ascribe to that the C is presented in the cationic part (PS[C_4mim]$^+$), which is hardly influenced by the conversion reaction and washing process.
